# A host of messages: *trans*-species micro-RNAs from the parasitic plant *Cuscuta campestris* share a common promoter

**DOI:** 10.1093/plcell/koad081

**Published:** 2023-03-17

**Authors:** Marco Bürger

**Affiliations:** Assistant Features Editor, The Plant Cell, American Society of Plant Biologists, USA; Plant Biology Laboratory, Salk Institute for Biological Studies, La Jolla, CA, USA

About 1% of all flowering plants are parasitic and break the “rules” of the plant world by relying on host plants for nutrients instead of photosynthesis. The stem-parasitic plant *Cuscuta campestris*, also known as field dodder, parasitizes more than 100 different plant species by attaching to them and penetrating their stems through a specialized organ, the haustorium. However, host plants often raise multiple defense mechanisms to prevent parasitic intrusion, and *Cuscuta* uses regulatory micro-RNAs (miRNAs) as one strategy to evade these defenses. These *trans*-species miRNAs are induced by *Cuscuta* at the parasite-host interface to target the host's immunity, development, and other signaling pathways (Shahid *et al*. 2018; Johnson *et al*. 2019). It is known that these miRNAs increase *Cuscuta*'s fitness, but their transcriptional regulation is still a mystery.

In a new breakthrough report, Collin Hudzik and colleagues (Hudzik *et al*. 2023) shed light on the origin of these *trans*-species miRNAs. By data mining recently published improved assemblies of the *C. campestris* genome and RNA-seq data sets, they annotated 156 interface-induced *MIRNA* loci. To understand when miRNA production occurs, Hudzik and colleagues performed small RNA-sequencing over a 15-day period and found that miRNA accumulation begins in the early stages of haustorium development before the host tissue is penetrated. This was observed in both Arabidopsis (*Arabidopsis thaliana*) and tomato (*Solanum lycopersicum*) hosts, indicating that miRNA induction might be host independent. To follow up on this question, the authors compared the levels of miRNA accumulation for each *MIRNA* locus in Arabidopsis and tomato hosts. The levels turned out to be quite similar, corroborating the idea that the induction of miRNA does not vary between different host species. Strikingly, miRNAs were also abundant in in vitro-generated haustoria that were not attached to any host, demonstrating that miRNA production is a developmental program rather than host-dependent.

A subsequent analysis of genomic sequences revealed a highly overrepresented 10-nucleotide motif adjacent to most of the interface-induced *MIRNA* loci, which was named “Upstream Sequence Element” (USE; see Fig 1.). Interestingly, the USE is identical to a known *cis*-regulatory element that drives U-snRNA transcription in plants (Vankan and Filipowicz, 1989) and has features consistent with transcription by RNA polymerase III. To test whether the USE is a *cis*-acting factor that promotes miRNA accumulation, the researchers designed *Agrobacterium tumefaciens* T-DNA vectors with *C. campestris* interface-induced *MIRNA* loci. When infiltrated into *Nicotiana benthamiana* leaves, the presence of intact loci resulted in mature miRNA accumulation, while scrambled loci did not.

**Figure 1.**
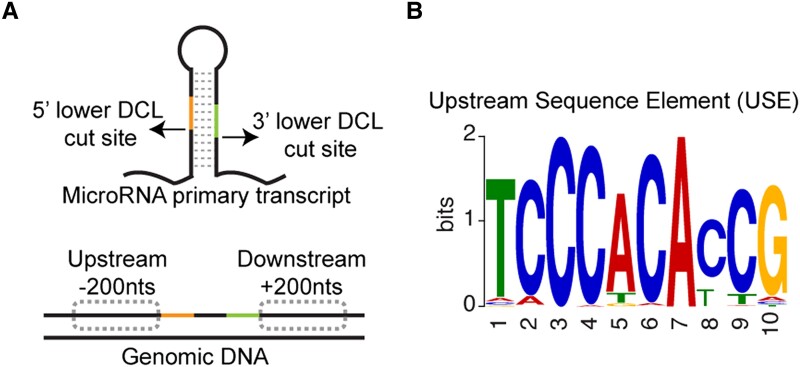
Interface-induced *MIRNA* loci share a common *cis*-regulatory element. **A**) Structure of the *MIRNA* primary transcript and genomic locus and **B**) the identified common USE sequence located upstream of the loci. Adapted from Hudzik et al. (2023), Figure 4.

This study provides great new insight into the genetic mechanisms of *C. campestris*' host adaptation and survival. It will be exciting to see if the USE is a common feature among parasitic plants and if it can serve as a potential strategy for reducing their virulence by targeting the USE-binding factors.

## References

[koad081-B1] Hudzik C , Maguire S, Guan S, Held J, and Axtell MJ. (2023). *Trans*-species microRNA loci in the parasitic plant *Cuscuta campestris* have a U6-like snRNA promoter. Plant Cell. 35(6): 1834–1847. 10.1093/plcell/koad076 PMC10226579 36896651

[koad081-B2] Johnson NR , dePamphilis CW, Axtell MJ. (2019). Compensatory sequence variation between trans-species small RNAs and their target sites. *Elife* 8:e49750. 10.7554/eLife.49750 PMC6917502 31845648

[koad081-B3] Shahid, S., Kim, G., Johnson, N.R., Wafula, E., Wang, F., Coruh, C., Bernal-Galeano, V., Phifer, T., dePamphilis, C.W., Westwood, J.H ., et al (2018). MicroRNAs from the parasitic plant Cuscuta campestris target host messenger RNAs. Nature. 553(7686): 82–85. 10.1038/nature25027 29300014

[koad081-B4] Vankan P , Filipowicz W. A U-snRNA gene-specific upstream element and a -30 ‘TATA box’ are required for transcription of the U2 snRNA gene of Arabidopsis thaliana. EMBO J. 1989:8(12):3875–3882. 10.1002/j.1460-2075.1989.tb08566.x 2583119 PMC402076

